# Healing of acute anterior cruciate ligament rupture on MRI and outcomes following non-surgical management with the Cross Bracing Protocol

**DOI:** 10.1136/bjsports-2023-106931

**Published:** 2023-06-14

**Authors:** Stephanie R Filbay, Matthew Dowsett, Mohammad Chaker Jomaa, Jane Rooney, Rohan Sabharwal, Phil Lucas, Andrew Van Den Heever, James Kazaglis, Justin Merlino, Mick Moran, Maggie Allwright, Donald E K Kuah, Ra Durie, Greg Roger, Mervyn Cross, Tom Cross

**Affiliations:** 1 Department of Physiotherapy, The University of Melbourne, Melbourne, Victoria, Australia; 2 School of Medicine, Sydney Campus, The University of Notre Dame Australia, Darlinghurst, New South Wales, Australia; 3 School of Public Health, University of Sydney Medical School, Sydney, New South Wales, Australia; 4 Lifecare Prahran Sports Medicine Centre, Melbourne, Victoria, Australia; 5 PRP Diagnostic Imaging, Sydney, New South Wales, Australia; 6 Stadium Sports Physiotherapy, Sydney, New South Wales, Australia; 7 Northern Beaches Hospital, Sydney, New South Wales, Australia; 8 New South Wales Institute of Sports, Sydney, New South Wales, Australia; 9 Sportsmed Manawatu, Palmerston North, New Zealand; 10 The University of Sydney School of Biomedical Engineering, Darlington, New South Wales, Australia; 11 Vestech Medical Pty Ltd, Sydney, New South Wales, Australia; 12 The Stadium Sports Medicine Clinic, Sydney, New South Wales, Australia

**Keywords:** Anterior Cruciate Ligament, Rehabilitation, Knee injuries, Physical Therapy, Orthopedics

## Abstract

**Objective:**

Investigate MRI evidence of anterior cruciate ligament (ACL) healing, patient-reported outcomes and knee laxity in patients with acute ACL rupture managed non-surgically with the Cross Bracing Protocol (CBP).

**Methods:**

Eighty consecutive patients within 4 weeks of ACL rupture were managed with CBP (knee immobilisation at 90° flexion in brace for 4 weeks, followed by progressive increases in range-of-motion until brace removal at 12 weeks, and physiotherapist-supervised goal-oriented rehabilitation). MRIs (3 months and 6 months) were graded using the ACL OsteoArthritis Score (ACLOAS) by three radiologists. Mann-Whitney U tests compared Lysholm Scale and ACL quality of life (ACLQOL) scores evaluated at median (IQR) of 12 months (7–16 months) post-injury, and χ^2^ tests compared knee laxity (3-month Lachman’s test and 6-month Pivot-shift test), and return-to-sport at 12 months between groups (ACLOAS grades 0–1 (continuous±thickened ligament and/or high intraligamentous signal) versus ACLOAS grades 2–3 (continuous but thinned/elongated or complete discontinuity)).

**Results:**

Participants were aged 26±10 years at injury, 39% were female, 49% had concomitant meniscal injury. At 3 months, 90% (n=72) had evidence of ACL healing (ACLOAS grade 1: 50%; grade 2: 40%; grade 3: 10%). Participants with ACLOAS grade 1 reported better Lysholm Scale (median (IQR): 98 (94–100) vs 94 (85–100)) and ACLQOL (89 (76–96) vs 70 (64–82)) scores, compared with ACLOAS grades 2–3. More participants with ACLOAS grade 1 had normal 3-month knee laxity (100% vs 40%) and returned to pre-injury sport (92% vs 64%), compared with participants with an ACLOAS grades 2–3. Eleven patients (14%) re-injured their ACL.

**Conclusion:**

After management of acute ACL rupture with the CBP, 90% of patients had evidence of healing on 3-month MRI (continuity of the ACL). More ACL healing on 3-month MRI was associated with better outcomes. Longer-term follow-up and clinical trials are needed to inform clinical practice.

**WHAT IS ALREADY KNOWN ON THIS TOPIC?**

- Poor long-term outcomes after anterior cruciate ligament (ACL) rupture are common following surgical or non-surgical management strategies, which are based on the assumption that a ruptured ACL has limited healing capacity
- A recent analysis of the KANON Trial found that at least 30% of participants with ACL rupture randomised to initial rehabilitation and optional surgery had signs of ACL healing (a continuous ACL) on a 2-year MRI.
- It is not clear whether a novel bracing protocol designed to facilitate healing of ACL rupture can improve patient outcomes following ACL rupture.

**WHAT ARE THE FINDINGS?**

- After management with the Cross Bracing Protocol (CBP), 72 out of 80 (90%) participants with complete discontinuity of the ACL at baseline had signs of ACL healing (ACL continuity) on 3-month MRI.
- Six out of eight ACLs that did not heal, had attached to the lateral wall±posterior cruciate ligament on a 3-month MRI.
- A lower ACL OsteoArthritis Score grade (more healing) on a 3-month MRI was associated with better self-reported knee function and knee-related quality of life, higher return to sport rates and reduced knee laxity.

**HOW MIGHT IT IMPACT ON CLINICAL PRACTICE IN THE FUTURE?**

- Considering the scarcity of research related to ACL healing and the novelty of the CBP, this study provides initial results to guide further research, including clinical trials.
- This study provides further evidence of the healing potential of the ACL, and the association between ACL healing on MRI and favourable patient outcomes.

## Background

A common belief among researchers and clinicians is that a ruptured anterior cruciate ligament (ACL) has limited healing capacity. This belief has shaped current management strategies for ACL rupture. However, anatomical studies have demonstrated that the ACL has a rich vascular supply^1 2^ and histological studies describe ruptured ACLs passing though the typical phases of healing after injury, despite a slower rate of healing and reduced healing capacity compared with medial collateral ligament rupture.^3–5^ An absence of tissue bridging the gap between ligament remnants has been observed, which may inhibit healing of ACL rupture.^6^ The distance between the ACL origin to its insertion is shortest at 90°–135° of knee flexion.^7^ We have developed the novel Cross Bracing Protocol (CBP) that aims to reduce the gap distance between the ligament remnants by immobilising the knee at 90° of flexion for 4 weeks after acute ACL rupture in attempt to facilitate bridging of tissue and healing between the ruptured ACL remnants. After 4 weeks, knee range-of-motion is increased at weekly increments and the CBP is coupled with physiotherapist-supervised rehabilitation targeting lower limb neuromuscular control, muscle strengthening and power, and functional training to enable return-to-sport and recreational activities.

A 2021 systematic review identified only six studies that evaluated ACL healing on MRI after ACL rupture.^8^ Studies were of low methodological quality and five studies included ≤50 participants.^8^ More recently, an analysis of the KANON Trial observed MRI evidence of ACL healing at a 2-year follow-up in 30% of participants who were randomised to initial rehabilitation and optional delayed ACL reconstruction (ACLR).^9^ Those with MRI evidence of ACL healing reported better 2-year knee function and quality of life (QOL), compared with participants with no MRI evidence of ACL healing, and participants managed with early or delayed ACLR.^9^ Many ACL injured people experience poor long-term outcomes, including sport and activity limitations, persistent pain, an early onset of osteoarthritis and poor long-term QOL.^10–13^ Considering the suboptimal outcomes with current management strategies, and the potential for ACL healing to result in favourable outcomes, new strategies to preserve and heal the native ACL should be explored. The objective of this study was to investigate MRI evidence of ACL healing, patient-reported outcomes and knee laxity in the first 80 individuals with acute ACL rupture managed non-surgically with the CBP.

## Methods

### Study design

This case series investigates outcomes from 80 consecutive patients with acute ACL rupture who were managed with the CBP. Data were collected in the course of clinical practice, and all participants provided informed consent for their data to be included in this study.

### Participants

**Figure 1:**
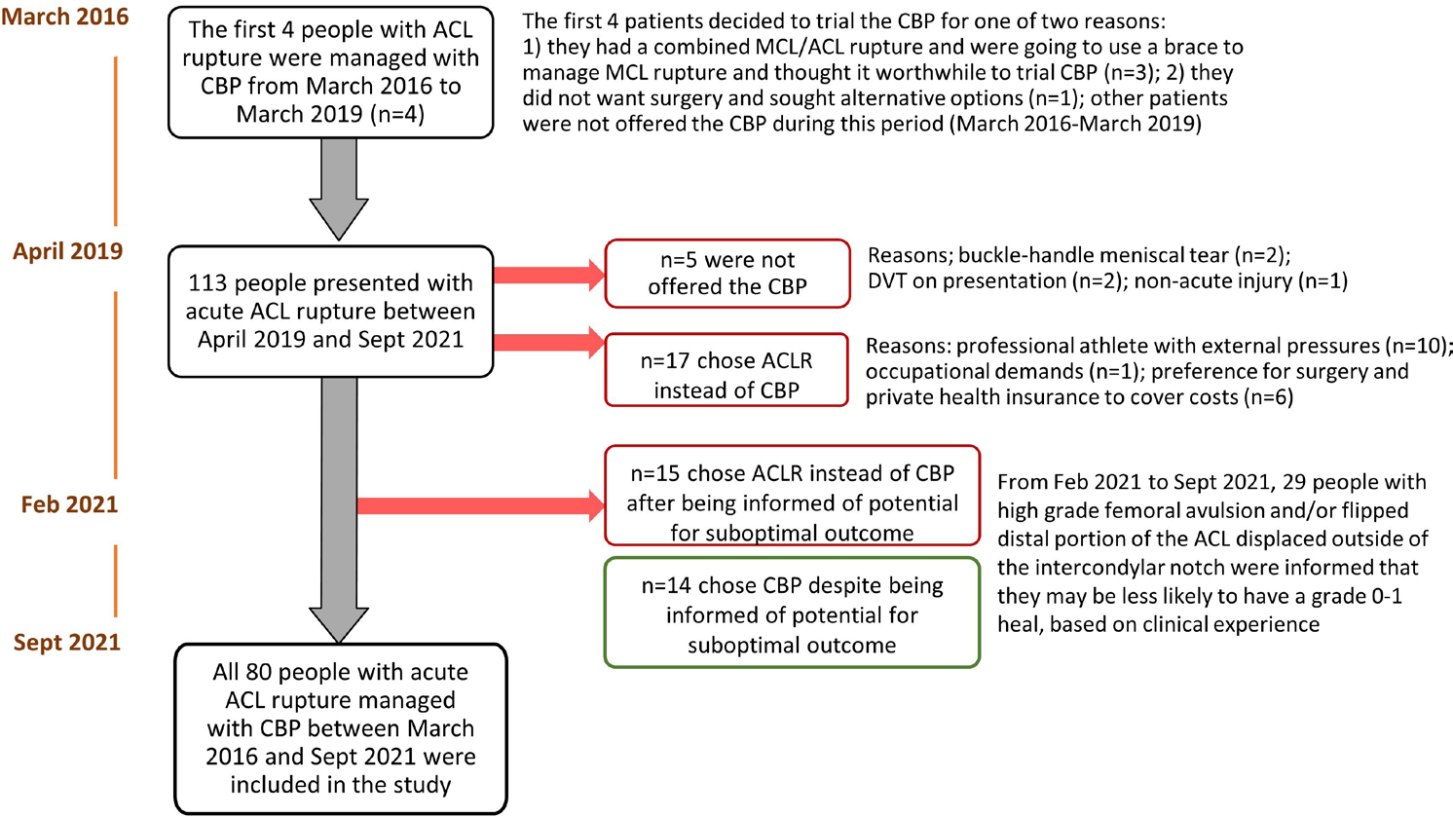
Participant flow chart. ACL, anterior cruciate ligament; ACLR, anterior cruciate ligament reconstruction; CBP, Cross Bracing Protocol; DVT, deep vein thrombosis; MCL, medial collateral ligament.

Eighty patients between the ages of 10 years and 58 years (mean (SD): 26 years (10 years)), who presented to a private sport and exercise medicine physician in Sydney, Australia (TC), with MRI confirmed acute ACL rupture between March 2016 and September 2021, were managed with the CBP (figure 1). Twelve out of 80 patients were residing outside of Sydney or impacted by COVID-19 restrictions and underwent virtual specialist consultations (TC), supplemented by in-person management and assessment from an experienced sports and exercise physician and physiotherapists trained in the CBP.

Patients of all ages, were considered eligible for the CBP if they presented within 1 month of acute ACL rupture, confirmed on MRI (ie, an ACL OsteoArthritis Score (ACLOAS) grade 3 representing full discontinuity of the ACL). To be considered for the CBP, patients needed to be functionally independent and capable of managing a period of knee immobilisation. Patients were considered ineligible if they had clinical or MRI evidence of structural concomitant injuries that necessitated surgical intervention (eg, an unstable bucket-handle meniscal tear) or a history of deep vein thrombosis (DVT) or pulmonary embolism. After the 10th participant, DVT screening was added to the eligibility criteria, whereby all patients underwent Doppler ultrasound to exclude DVT. The flow of participants through the study, including reasons for not offering the CBP and reasons for choosing ACLR (no participants chose rehabilitation alone), is presented in figure 1.

### Management decision

Patients were informed about treatment options: early ACLR, non-operative rehabilitation with optional delayed ACLR or trialling the CBP. Patients received information on the rationale and theoretical justification for the CBP, and they were aware that this was an experimental treatment with a chance of failure and possible need for ACLR in the future. The decision to trial the CBP was based on patient preference.

### Cross Bracing Protocol

The CBP and accompanying rehabilitation protocol is described in online supplemental appendix 1. In patients presenting in the first week post-injury, the use of cryotherapy and anti-inflammatory medications was discouraged to minimise impairment of the acute inflammatory response.^5 14^ Paracetamol was prescribed as needed for pain. Fourteen patients who presented ≥7 days post-injury with minimal or no hemarthrosis/effusion underwent a platelet-rich plasma injection.

10.1136/bjsports-2023-106931.supp1 Supplementary data

The injured knee was then secured at 90° flexion in a standard limited range-of-motion brace as early as convenient following injury (range: 0–31 days post-injury, median (IQR): 5 days (4–11 days)), by a physiotherapist trained in the CBP. Patients were advised to keep the knee fixed in the brace at all times for the first 4 weeks, including during sleep and showering. Patients were educated regarding safe use of crutches during the first 8 weeks (and use of additional mobility aids if desired, such as a knee scooter, i-Walker or wheelchair), while unable to sufficiently extend the knee to walk unaided. Patients were given advice regarding self-care, comfort and DVT risk mitigation strategies, including hydration and calf pump exercises. Prophylactic Clexane injections were introduced after the 10th patient. From patient 20 onwards, Rivaroxiban 10 mg was prescribed (for the first 8 weeks of the CBP) instead of Clexane.

After 4 weeks, the range-of-motion brace was adjusted at regular increments to allow progressive increases in range-of-motion (see online supplemental appendix 1). At week 10, unrestricted range-of-motion was allowed, and the brace was removed at 12 weeks. Weight-bearing was encouraged within the available range and patients completed standardised goal-oriented exercise-based rehabilitation while in the brace, and after brace removal until the point of return-to-sport (online supplemental appendix 1). Patients had weekly physiotherapist consults to check/adjust the brace and progress exercise-based rehabilitation (online supplemental appendix 1). Return-to-sport was not recommended until 9–12 months post-injury, and was dependent on patient and clinical factors, including desire to return-to-sport, completion of required rehabilitation and passing functional return-to-sport criteria.^15^

### Deviations from protocol

The first 4 patients had the brace removed and their first follow-up assessment at 9 weeks. After this, a decision was made to extend the CBP from 9 weeks to 12 weeks to protect the ACL for longer and enable more accurate interpretation of the MRI at the time of brace removal. Additionally, two patients for personal reasons (work demands/to care for young children) removed the brace at the end of week 4 and week 6. Both patients were compliant with the CBP before brace removal and completed rehabilitation post brace removal.

### Outcomes

Details of all outcomes, including measurement method, time of measurement and interpretation, are presented in table 1.

**Table 1:** Outcome measurement and interpretation

Construct (measure)	Time-point(s)	Assessment method	Interpretation
Evidence of ACL healing on MRI (ACLOAS MRI grading system)^21^	3 months; 6 months	MRI interpretation was performed independently by three experienced musculoskeletal radiologists (RS, PL and AVDH). A consensus meeting resolved any discrepancies	ACLOAS grades: 0=normal ligament with hypointense signal and regular thickness and continuity; 1=thickened ligament and/or high intraligamentous signal with normal course and continuity; 2=thinned or elongated but continuous ligament; 3=absent ligament or complete discontinuity^21^
Self-reported knee function (Lysholm Scale)^22^	Median (IQR): 12 months (7–16 months)	Self-reported questionnaire	Scored out of 100 across 8 domains: pain, instability, locking, swelling, limping, stair climbing, squatting and the need for gait support.^23^ A score of 84 is considered ‘good’. A score of >94 is considered ‘excellent’. A lower score indicates increased severity of symptoms and worse function.^24^ Acceptable reliability, validity and responsiveness for use in ACL-injured individuals^25^
Knee-related QOL (ACLQOL)^26^	Median (IQR): 12 months (7–16 months)	Self-reported questionnaire	The 32-item questionnaire contains five domains (Symptoms & Physical Complaints, Work-Related Concerns, Recreation Activities & Sport Participation or Competition, Lifestyle, Social & Emotional Aspects) and is scored out of 100 (overall score and individual domain scores) with a lower score indicating worse knee-related QOL.^26^ Valid, reliable and responsive for use in ACL injured patients^17 27^
Passive knee laxity (Lachman’s test and pivot- shift test)	3 months (Lachman’s test); 6 months (pivot-shift test)	Both tests were performed by one of two experienced sports and exercise medicine physicians (TC and RD) who were not blinded to the treatment	Lachman’s test was graded relative to the contralateral knee (no side-to-side difference; end-point with side-to-side difference and no end-point).^28^ Pivot shift test was graded based on the quality of the lateral pivot-shift manoeuvre (normal=no glide or jerk detected; glide=a ‘gliding’ motion (IKDC grade 1^29^); jerk=locked subluxation (described as grade 3 instability by the IKDC classification^29^); or a ‘jerking’/clunking movement pattern (IKDC grade 2 instability^29^))
Return to pre-injury sport	12 months	Self-reported during a clinical visit or via telephone interview	Patients reported whether they had returned to pre-injury sport (information collected included the type and level of sport that they returned to)

ACL, anterior cruciate ligament; ACLOAS, Anterior Cruciate Ligament OsteoArthritis Score; ACLQOL, Anterior Cruciate Ligament Quality Of Life Questionnaire; IKDC, International Knee Documentation Committee; QOL, quality of life.

### Patient and public involvement

Patients have been involved in the development and refinement of the CBP, and the authorship team includes a patient who was managed with the CBP (MD).

### Equity, diversity and inclusion

All patients with acute ACL rupture managed with the CBP prior to October 2021 participated in the study, including 31 (39%) females, people aged 10–58 years at the time of injury, and both private (69%) and publicly (31%) funded patients. Although only 3 women are included in the authorship team, the lead researcher is a woman and we include authors from a variety of career stages and clinical disciplines.

### Statistical analysis

All continuous variables were assessed for normality and mean (SD) or median (IQR) reported, as appropriate. Participant characteristics and outcomes are presented for all participants, and based on 3-month ACLOAS. Mann-Whitney U tests were used to compare continuous outcomes (Lysholm Scale and ACLQOL scores) and Pearson’s χ^2^ tests were used to compare categorical outcomes (Lachman’s test, Pivot-shift test and return-to-sport) between groups with lower versus higher ACLOAS grades on 3-month MRI (ACLOAS grades 0–1 vs ACLOAS grades 2–3). Since seven participants completed the Lysholm Scale and ACLQOL score after ACL re-rupture, a subgroup analysis was performed to present data and compare groups after excluding these seven individuals from the analysis (online supplemental appendix 2). For the two participants with missing MRI data at 3-month follow-up (decided not to undergo MRI), the ACLOAS from 6-month MRI was used to classify 3-month ACLOAS for analysis (95% of participants had the same ACLOAS at 3 months and 6 months, only 1 participant had a worse ACLOAS grade due to re-injury). Six people were missing 6-month MRI data (due to ACL re-rupture (n=3), pregnancy (n=1) or decided not to undergo MRI (n=2)). Since only one participant had missing data for the Lysholm Scale and ACLQOL scores, a complete case analysis was performed.

10.1136/bjsports-2023-106931.supp2 Supplementary data

## Results

### Participant characteristics

All individuals managed with the CBP provided consent for their data to be included in this study. Participants were aged a mean (SD) 26 (10) years at injury, 39% were female and 49% had concomitant meniscal injury (38 stable vertical tears in posterior horn of medial and/or lateral meniscus and 1 displaced medial meniscus ramp lesion). Participant characteristics are reported in table 2 for all participants and by ACLOAS grade on 3-month MRI (grade 1 vs grades 2–3). Participant characteristics are presented separately for participants with an ACLOAS grades 2 and 3, in online supplemental appendix 3.

10.1136/bjsports-2023-106931.supp3 Supplementary data

**Table 2:** Participant characteristics

	All participants (n=80)	Evidence of ACL healing on 3 month MRI	
		ACLOAS grade 1 (n=40)	ACLOAS grades 2–3 (n=40)
Age at injury (SD)	26 (10)	27 (10)	26 (10)
Sex (% female)	31 (39)	14 (35)	16 (40)
Time from injury to brace (days)	8 (7)	6 (4)	8 (7)
Used private health insurance	55 (69)	27 (68)	27 (68)
Level of pre-injury sport			
Recreational	28 (35)	13 (33)	15 (38)
Competitive	49 (61)	25 (63)	23 (58)
Professional	4 (5)	2 (5)	2 (5)
Contact mechanism of injury	24 (30)	17 (43)	7 (18)
History of contralateral ACL injury	8 (10)	3 (8)	5 (13)
Adherent to bracing protocol	77 (96)	37 (93)	39 (98)
PRP injection	14 (18)	5 (13)	9 (23)
Concomitant injuries*			
MCL injury	40 (50)	17 (43)	23 (58)
Meniscal injury	39 (49)	21 (53)	18 (45)
PLC injury	31 (39)	20 (50)	11 (28)
Bone contusion	74 (93)	38 (95)	35 (88)
Chondral injury	1 (1)	1 (3)	0 (0)
Subcortical fracture	6 (8)	3 (8)	3 (8)
ACL rupture characteristics*			
ACL femoral origin intact:	36 (45)	33 (83)	3 (8)
Displacement of ACL tissue†	17 (21)	14 (35)	2 (5)
Partial avulsion of femoral origin:	44 (55)	7 (18)	37 (93)
Displacement of ACL tissue†	33 (41)	4 (10)	29 (73)
Complete avulsion of femoral origin	0 (0)	0 (0)	0 (0)

Data are reported as mean (SD) or count (proportion).

For the two participants with missing 3-month MRI data, ACLOAS was estimated using ACLOAS from 6-month MRI (70 out of 75 (93%) participants with 6-month MRI data had the same ACLOAS at 3 months and 6 months).

*Concomitant injuries and ACL rupture characteristics were assessed by MRI within 3 weeks of acute ACL rupture.

†ACL tissue is displaced outside the boundaries of the intercondylar notch;.

ACLOAS, Anterior Cruciate Ligament OsteoArthritis Score; MCL, medial collateral ligament; PLC, posterior lateral corner; PRP, platelet rich plasma.

### ACL healing as visualised on MRI

**Figure 2:**
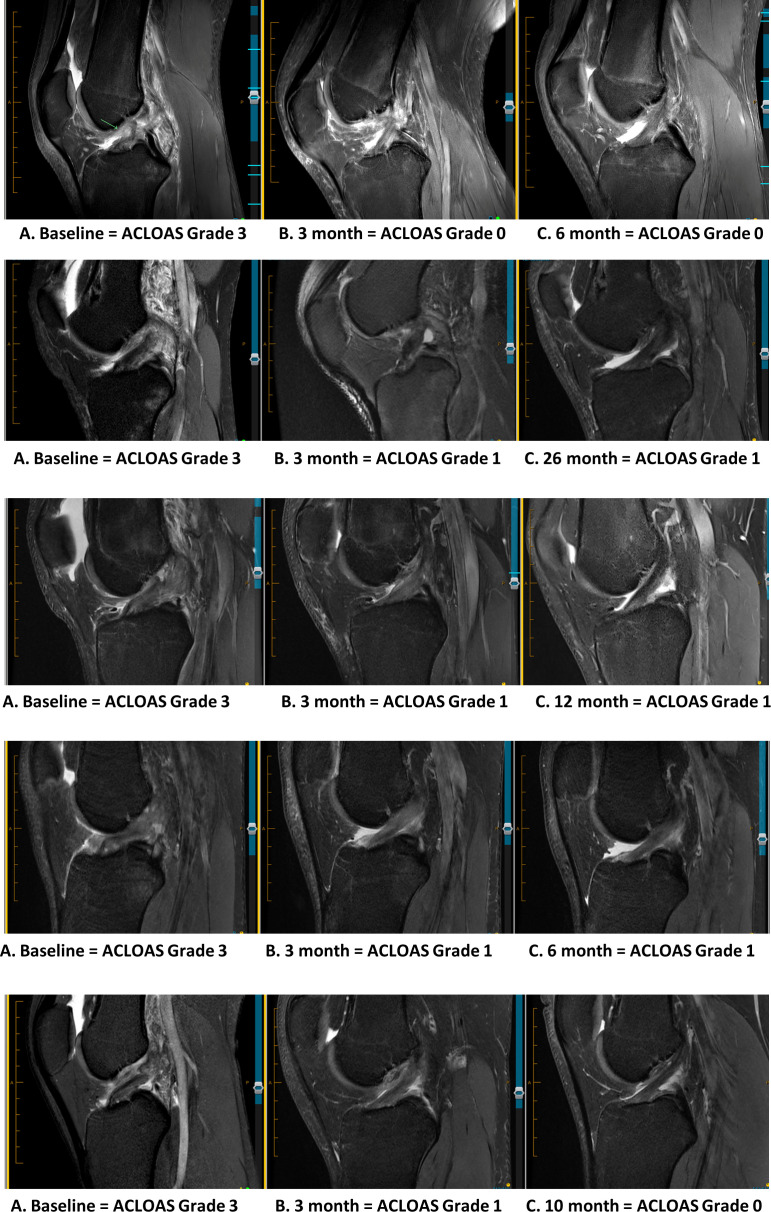
MRI images demonstrating MRI evidence of ACL healing for five participants. ACL, anterior cruciate ligament; ACLOAS, Anterior Cruciate Ligament OsteoArthritis Score.

At 3-month follow-up, n=72 (90%) had a continuous ACL (n=40 (50%) ACLOAS grade 1, n=32 (40%) ACLOAS grade 2). Of the 8 patients with ACLOAS grade 3 on 3-month MRI, 6 ACLs had attached to the lateral wall (n=3) or lateral wall and posterior cruciate ligament (n=3). Between 3-month and 6-month MRI, 4 participants changed from ACLOAS grade 1 to grade 0 and 1 participant changed from ACLOAS grade 2 to grade 3 due to subsequent knee injury. ACLOAS grades from 3-month and 6-month MRIs (complete case analysis) are presented in online supplemental appendix 4. Other participants sustained the same ACLOAS grade at 3 months and 6 months. MRI examples of ACL healing for five participants are presented in figure 2.

10.1136/bjsports-2023-106931.supp4 Supplementary data

### Patient-reported outcomes and passive knee laxity

Participants with an ACLOAS grade 1 on 3 month MRI reported better patient-reported outcomes on the Lysholm Scale and ACLQOL (including all ACLQOL subscales) compared with participants with an ACLOAS grades 2–3 (table 3). Online supplemental appendix 2 presents outcomes separately for participants with an ACLOAS grades 2 and 3. Online supplemental appendix 5 depicts participant scores based on time post-injury and 3-month healing status. Participants with an ACLOAS grade 1 had reduced knee laxity and a higher proportion returned to pre-injury sport (92% vs 62%) compared with participants with an ACLOAS grades 2–3 (table 3).

10.1136/bjsports-2023-106931.supp5 Supplementary data

**Table 3:** Participant outcomes

	All participants (n=80)	Evidence of ACL healing on 3-month MRI		P value*
		ACLOAS grade 1 (n=40)	ACLOAS grades 2–3 (n=40)	
Lysholm Scale score	95 (89 to 100)	98 (94 to 100)	94 (85 to 94)	0.01
ACLQOL score (total)	80 (69 to 93)	89 (76 to 96)	70 (64 to 82)	<0.001
Symptoms/physical complaints	94 (86 to 98)	96 (92 to 100)	86 (82 to 96)	<0.001
Work-related concerns	98 (90 to 100)	100 (98 to 100)	95 (79 to 100)	0.004
Rec and sport participation	68 (54 to 91)	82 (65 to 93)	58 (42 to 78)	0.001
Lifestyle	88 (74 to 98)	94 (86 to 100)	78 (63 to 93)	<0.001
Social and emotional	78 (58 to 94)	90 (72 to 94)	68 (52 to 80)	0.003
3-month Lachman’s test				
No SSD	64 (80)	40 (100)	24 (60)	<0.001
End point with SSD	15 (19)	0 (0)	15 (38)	
No endpoint	1 (1)	0 (0)	1 (3)	
6-month pivot-shift test				
Normal	44 (58)	33 (85)	11 (30)	<0.001
Glide	31 (41)	6 (15)	25 (68)	
Jerk	1 (1)	0 (0)	1 (3)	
Missing, n	4	1	3	
Return to pre-injury sport at 12 months				
Yes	59 (79)	36 (92)	23 (62)	<0.001
No	16 (21)	3 (8)	13 (35)	
N/A (early cross-over to ACLR)	5	1	4	
Underwent arthroscopic surgery	2 (3)	1 (3)	1 (3)	1.0
ACL re-rupture	11 (14)	4 (10)	7 (18)	0.17

For n=2 who did not have an MRI at 3 months, 6 month MRI results are reported (93% of participants had the same ACLOAS at 3 months and 6 months).

Missing data from n=1 for the Lysholm Scale and ACLQOL (ACLOAS grade 2).

Non-normally distributed data are presented as median (IQR).

*Mann-Whitney U Test (continuous variables) or Pearson’s χ^2^ test (categorical variables) compared outcomes between groups (ACLOAS grade 1 vs ACLOAS grades 2–3), the p value represents the probability of obtaining the observed results assuming that the null hypothesis is true.

ACLOAS, Anterior Cruciate Ligament OsteoArthritis Score; ACLR, anterior cruciate ligament reconstruction; N/A, not applicable; RTS, return-to-sport; SSD, side to side difference.

### ACL re-injury and subsequent surgery

Eleven (14%) participants re-injured their ACL (mean (SD): 10 months (4 months), range: 5–18 months), 4 had ACLOAS grade 1, and 7 had ACLOAS grade 2 on 3-month MRI. After re-injury, 9 of 11 participants underwent ACLR (mean: 2 months after re-injury, range: 0–6 months), 1 participant decided to undergo the CBP again (resulting in evidence of ACL healing on MRI, ACLOAS grade 1). Mechanisms of re-injury included AFL/rugby (n=3), basketball (n=1), skiing (n=1), cycling accident (n=1), netball (n=1), Oz-tag (n=1), wrestling (n=1), dancing (n=1) and climbing (n=1). The four participants who re-injured their ACL despite ACLOAS grade 1 on 3-month MRI, did so during high-speed skiing/cycling accidents (5 months and 18 months post-injury), rugby (contact injury 10 months post-injury) and AFL (contact injury 17 months post-injury).

Two of 80 (2.5%) participants underwent an arthroscopic knee surgery, one participant for cyclops lesion removal 7 months post-injury and another underwent a partial lateral meniscectomy 5 months post-injury. Thirty eight of 39 (97%) meniscal tears were asymptomatic following the CBP, including one displaced medial meniscus ramp lesion.

### Adverse events

Two patients were diagnosed with a below knee DVT (before DVT prophylaxis was added to the protocol), which were identified in the second week of the CBP, and were successfully managed with therapeutic dosing of Clexane. Both patients completed the CBP. Follow-up Doppler ultrasound demonstrated complete resolution of the DVT for both patients. Thereafter (11th patient onwards), DVT risk mitigation strategies were deployed as described in the Methods section.

Most patients reported mild and transient discomfort while adapting to the brace during the first week, often citing an awkward or uncomfortable sleeping position with the knee fixed at 90°. This discomfort resolved for all patients without intervention. No patients opted to exit the programme due to discomfort or complication. At the time of unrestricted knee flexion in the brace, a flexion contracture (typically 5°–15°) was observed in 11 patients (14%). This resolved in all patients with physiotherapy exercises within 3 weeks. Contralateral lower limb overuse injuries, including pes anserine bursitis (n=1), insertional hamstrings tendinopathy (n=1) and patellofemoral pain (n=3), were observed at the time of brace removal.

## Discussion

### Summary

This case series found that 72 out of 80 (90%) people with acute ACL rupture who were managed with a novel bracing protocol involving immobilisation of the knee at 90° flexion, had evidence of ACL healing (a continuous ACL) on 3-month MRI. An ACLOAS grade 1 on 3-month MRI was associated with better 12-month knee function and QOL, reduced passive knee laxity and a higher rate of return-to-sport, compared with an ACLOAS grades 2–3.

### ACL healing

A recent analysis of the KANON trial found that 16 of 54 (30%) participants randomised to initial rehabilitation and optional delayed ACLR had signs of ACL healing on a 2-year MRI.^9^ Of the 30 participants who were managed with rehabilitation alone, 53% had MRI evidence of ACL healing at 2 years.^9^ In comparison, applying the same criteria we observed ACL healing in 72 of 80 (90%) participants on 3-month MRI. Six of 8 ACLs with discontinuous fibres had attached to the lateral wall±posterior cruciate ligament. Although we graded these as ‘discontinuous’, it is possible that attachment to these structures could provide some function/stability, and it is not clear how this compares to the function/stability of an ACL graft. The high rate of healing observed on 3-month MRI suggests that the CBP could be conducive to ACL healing. To explore this potential, further research, including mechanistic studies, is required. Interestingly, patients had a range of concomitant injuries at baseline which became asymptomatic after the CBP. Only 1 of 39 patients with concomitant meniscal injuries had persistent symptoms after the CBP and underwent meniscal surgery. It is possible that the CBP could be beneficial for healing of concomitant injuries, this warrants further research.

Additionally, 37 of 40 participants (93%) with an ACLOAS grades 2–3 on 3-month MRI had an ACL rupture with a partial femoral avulsion, compared with only 7 (18%) participants with an ACLOAS grade 1. Although outside the scope of this study, it is possible that characteristics of ACL rupture observed on acute MRI (including partial/complete femoral avulsion, the displacement of ACL tissues outside of the intercondylar notch and gap distance between the ruptured ACL stumps) are associated with the likelihood of ACL healing. Further studies are needed to explore this possibility, with potential to inform ACL injury management decisions.

### Interpretation of outcomes

The favourable outcomes observed in patients with signs of ACL healing in our study are supported by findings from the KANON trial. In the KANON trial, participants with an ACLOAS of 0–2 on a 2-year MRI reported better knee function and QOL compared with participants with ACL discontinuity, and people who had delayed or early ACLR.^9^ Notably, only 8 (10%) patients in our study had ACL discontinuity on a 3-month MRI, and we used a different cut-off when comparing outcomes between groups. Collectively, results from the KANON trial and CBP suggest there may be a spectrum of ACL healing, whereby a more ‘normal’ MRI appearance of the ACL may be associated with favourable patient outcomes.

Surprisingly, patients with an ACLOAS grades 2–3 reported excellent Lysholm Scale scores on average, even though scores were lower than patients with an ACLOAS grade 1. A Lysholm median score of 98 reported by people with an ACLOAS grade 1 is better than mean scores reported 24–71 months after ACLR using autograft (mean scores range from 85 to 95).^16^ In contrast, the difference in ACLQOL scores was large between people with lower and higher grades of ACL healing. People with an ACLOAS grade 1 reported a median (IQR) ACLQOL score of 89 (76–96). In comparison, people managed with ACLR or rehabilitation alone, report mean ACLQOL scores in the range of 50–76, across a variety of time-points after ACL injury.^17^ The ACLQOL scores reported by people with an ACLOAS grades 2–3 may be more comparable with ACLQOL scores reported after ACLR and management with rehabilitation alone.^17^ Examining the ACLQOL domain scores suggests the greatest differences were within the recreational and sport participation, lifestyle and social emotional domains, with smaller differences observed in the symptoms and physical complaints and work-related concern domains.

The lower proportion who returned to sport with an ACLOAS grades 2–3 (64%) compared with an ACLOAS grade 1 (92%) could partly explain the lower QOL in this group considering return-to-sport is a key determinant of QOL after ACL injury.^18^ These return-to-sport rates are high compared with studies in ACL reconstructed individuals, where a pooled average of 55% of non-professional athletes returned to sport after ACLR.^10^ It should also be noted that patients in our study were aware of the degree of healing observed on MRI. It is possible that patients who received feedback that they had a suboptimal healing result on MRI had lower knee confidence and negative mental impacts compared with patients who received more positive feedback. This could also contribute to lower QOL scores in these domains.

### Subsequent injury

It is not known whether a continuous ACL observed on MRI reflects restoration of pre-injury ACL function. Although, the high self-reported knee function and return-to-sport rate in people with an ACLOAS grade 1 suggests a positive association with knee function. It is important to note that 11 patients (14%) had re-ruptured their ACL at the time of follow-up. The 4 patients who re-injured their ACL despite an ACLOAS grade 1 on a 3-month MRI did so during competitive sport (rugby/AFL contact injuries) or high-speed skiing/cycling accidents. It is not clear whether re-injury of the ACL is a reflection of reduced tensile strength of the ACL fibres, considering the mechanisms of re-injury were similar to the mechanisms of initial ACL rupture. The rate of re-injury observed in our study may be comparable with re-injury rates following ACLR, whereby approximately 1-in-5 young athletes suffer a rupture of the ACL graft or contralateral ACL, and around 90% of these injuries occur after return to high-risk sports.^19^ Interestingly, one patient elected to undergo the CBP after re-rupturing their ACL and achieved an ACLOAS grade 1 on a 3-month MRI and returned to sport (rugby), after re-completion of the CBP. Longer-term follow-up is required to gain greater understanding of survivorship of the healed ACL and the risk of subsequent knee injury following management with the CBP.

### Implications of findings

In view of the promising outcomes of this case series and the potential advantages of preserving the native ACL following injury, further research in this area is warranted. Prognostic studies are needed to determine whether certain presentations are less likely to heal when managed with the CBP. In the future, the potential for the ACL to heal may be an important consideration when deciding on surgical or non-surgical management. We found that signs of ACL healing were apparent on MRI as early as 3 months after ACL rupture. Three months is the typical duration that people trial initial rehabilitation before considering delayed ACLR. It is possible that 3-month MRI findings could identify patients who would benefit from ACLR. Only 10% of patients with an ACLOAS grade 1 at 3 months had progressed to an ACLOAS grade 0 at 6 months. Further research is needed to understand the timeline and stages of ACL healing. Additionally, clinical trials are needed to compare outcomes for patients managed with the CBP, compared with those who are managed with ACLR or rehabilitation alone. Of particular importance will be the investigation of re-injury rates, return-to-sport, patient-reported outcomes, functional stability and the prevalence of knee osteoarthritis.

Bridge enhanced ACL repair (BEAR) is a surgical technique that augments repair of the ligament with a scaffold implant (a resorbable protein-based implant containing autologous blood) positioned between the torn ends of a midsubstance ACL tear in attempt to facilitate healing.^20^ An interesting area for future research is the comparison of ACL healing on MRI and clinical outcomes following management with BEAR compared with the CBP. Additionally, there may be specific ACL rupture presentations that benefit from early surgical intervention to assist with facilitating ACL healing. For example, acute ACL injuries with a large gap distance between torn remnants and acute ACL injuries with displaced ACL tissue outside the intercondylar notch might benefit from surgery to reduce and realign the ACL tissues. There could also be a role for bracing in knee flexion postoperatively to protect the repair and/or reduction of the injured ACL tissues (akin to management of displaced bone fractures with open reduction internal fixation followed by a period of postoperative immobilisation). Further research is needed to explore this potential.

### Limitations

This was a pragmatic study, whereby data were collected in the course of clinical practice rather than in a research setting. For this reason, some adaptations were made to the CBP overtime. Our study design did not allow comparison of outcomes with people managed with ACLR or rehabilitation alone. There is also potential for selection bias. After the 50th patient was braced, patients were discouraged from undertaking the CBP if they had a femoral avulsion and/or ACL tissue displaced outside the boundaries of the intercondylar notch. Although 14 of 29 patients underwent the CBP despite this advice, 15 of these individuals chose ACLR. The overall proportion with an ACLOAS grade 1 at 3 months may have been lower if these 15 individuals had not received this advice and underwent the CBP. Tests of passive knee laxity were performed by two unblinded physicians and may be subject to detection and observer bias, a clinical trial, including blinding of examiners, is needed. Further studies may also benefit from using a knee arthrometer to collect more objective measures of knee laxity. MRIs were graded by three radiologists who were aware that patients had undertaken the CBP. Although this case series highlights the potential for positive outcomes using the CBP, larger cohorts with longer-term follow-up and, in particular, randomised clinical trials are needed.

## Conclusion

After management of acute ACL rupture with a novel bracing protocol, 90% of patients had evidence of ACL healing on a 3-month MRI (continuity of the ACL). More ACL healing on a 3-month MRI was associated with better knee function and QOL, less passive knee laxity and a higher return-to-sport rate.

## Data Availability

Data are available upon reasonable request.

## References

[1] Scapinelli R . Vascular anatomy of the human cruciate ligaments and surrounding structures. Clin Anat 1997;10:151–62. 10.1002/(SICI)1098-2353(1997)10:3<151::AID-CA1>3.0.CO;2-X 9135883

[2] Toy BJ , Yeasting RA , Morse DE , et al . Arterial supply to the human anterior cruciate ligament. J Athl Train 1995;30:149–52. 16558326 PMC1317848

[3] Yoshida M , Fujii K . Differences in cellular properties and responses to growth factors between human ACL and MCL cells. J Orthop Sci 1999;4:293–8. 10.1007/s007760050106 10436277

[4] Nguyen DT , Ramwadhdoebe TH , van der Hart CP , et al . Intrinsic healing response of the human anterior cruciate ligament: an histological study of reattached ACL remnants. J Orthop Res 2014;32:296–301. 10.1002/jor.22511 24600702

[5] Witkowski J , Yang L , Wood DJ , et al . Migration and healing of ligament cells under inflammatory conditions. J Orthop Res 1997;15:269–77. 10.1002/jor.1100150217 9167631

[6] Murray MM , Martin SD , Martin TL , et al . Histological changes in the human anterior cruciate ligament after rupture. J Bone Joint Surg Am 2000;82:1387–97. 10.2106/00004623-200010000-00004 11057466

[7] Jordan SS , DeFrate LE , Nha KW , et al . The in vivo kinematics of the anteromedial and posterolateral bundles of the anterior cruciate ligament during weightbearing knee flexion. Am J Sports Med 2007;35:547–54. 10.1177/0363546506295941 17261571

[8] Pitsillides A , Stasinopoulos D , Giannakou K . Healing potential of the anterior cruciate ligament in terms of fiber continuity after a complete rupture: a systematic review. J Bodyw Mov Ther 2021;28:246–54. 10.1016/j.jbmt.2021.06.003 34776148

[9] Filbay SR , Roemer FW , Lohmander LS , et al . Evidence of ACL healing on MRI following ACL rupture treated with rehabilitation alone may be associated with better patient-reported outcomes: a secondary analysis from the KANON trial. Br J Sports Med 2023;57:91–9. 10.1136/bjsports-2022-105473 36328403 PMC9872245

[10] Ardern CL , Taylor NF , Feller JA , et al . Fifty-five per cent return to competitive sport following anterior cruciate ligament reconstruction surgery: an updated systematic review and meta-analysis including aspects of physical functioning and contextual factors. Br J Sports Med 2014;48:1543–52. 10.1136/bjsports-2013-093398 25157180

[11] Lie MM , Risberg MA , Storheim K , et al . What's the rate of knee osteoarthritis 10 years after anterior cruciate ligament injury? An updated systematic review. Br J Sports Med 2019;53:1162–7. 10.1136/bjsports-2018-099751 30936063

[12] Filbay SR , Ackerman IN , Russell TG , et al . Health-related quality of life after anterior cruciate ligament reconstruction: a systematic review. Am J Sports Med 2014;42:1247–55. 10.1177/0363546513512774 24318609

[13] Filbay SR , Culvenor AG , Ackerman IN , et al . Quality of life in anterior cruciate ligament-deficient individuals: a systematic review and meta-analysis. Br J Sports Med 2015;49:1033–41. 10.1136/bjsports-2015-094864 26224582

[14] Hubbard TJ , Denegar CR . Does cryotherapy improve outcomes with soft tissue injury J Athl Train 2004;39:278–9. 15496998 PMC522152

[15] Filbay SR , Grindem H . Evidence-based recommendations for the management of anterior cruciate ligament (ACL) rupture. Best Practice & Research Clinical Rheumatology 2019;33:33–47. 10.1016/j.berh.2019.01.018 31431274 PMC6723618

[16] Carey JL , Dunn WR , Dahm DL , et al . A systematic review of anterior cruciate ligament reconstruction with autograft compared with allograft. J Bone Joint Surg Am 2009;91:2242–50. 10.2106/JBJS.I.00610 19724004 PMC2730860

[17] Filbay SR , Grevnerts HT , Sonesson S , et al . The Swedish version of the anterior cruciate ligament quality of life measure (ACL-QOL): translation and measurement properties. Qual Life Res 2023;32:593–604. 10.1007/s11136-022-03265-1 36227526 PMC9911474

[18] Filbay SR , Ackerman IN , Russell TG , et al . Return to sport matters—longer-term quality of life after ACL reconstruction in people with knee difficulties. Scand J Med Sci Sports 2017;27:514–24. 10.1111/sms.12698 27167588

[19] Barber-Westin S , Noyes FR . One in 5 athletes sustain reinjury upon return to high-risk sports after ACL reconstruction: a systematic review in 1239 athletes younger than 20 years. Sports Health 2020;12:587–97. 10.1177/1941738120912846 32374646 PMC7785893

[20] Murray MM , Fleming BC , Badger GJ , et al . Bridge-enhanced anterior cruciate ligament repair is not inferior to autograft anterior cruciate ligament reconstruction at 2 years: results of a prospective randomized clinical trial. Am J Sports Med 2020;48:1305–15. 10.1177/0363546520913532 32298131 PMC7227128

[21] Roemer FW , Frobell R , Lohmander LS , et al . Anterior cruciate ligament osteoarthritis score (ACLOAS): longitudinal MRI-based whole joint assessment of anterior cruciate ligament injury. Osteoarthritis and Cartilage 2014;22:668–82. 10.1016/j.joca.2014.03.006 24657830

[22] Tegner Y , Lysholm J . Rating systems in the evaluation of knee ligament injuries. Clin Orthop Relat Res 1985;1985:43–9. 4028566

[23] Lysholm J , Gillquist J . Evaluation of knee ligament surgery results with special emphasis on use of a scoring scale. Am J Sports Med 1982;10:150–4. 10.1177/036354658201000306 6896798

[24] Collins NJ , Misra D , Felson DT , et al . Measures of knee function: International Knee Documentation Committee (IKDC) subjective knee evaluation form, Knee Injury and Osteoarthritis Outcome Score (KOOS), Knee Injury and Osteoarthritis Outcome Score Physical Function Short Form (KOOS-PS), Knee Outcome Survey Activities of Daily Living Scale (KOS-ADL), Lysholm knee scoring scale, Oxford Knee Score (OKS), Western Ontario and Mcmaster Universities Osteoarthritis Index (WOMAC), Activity Rating Scale (ARS), and Tegner Activity Score (TAS). Arthritis Care Res (Hoboken) 2011;63 Suppl 11:S208–28. 10.1002/acr.20632 22588746 PMC4336550

[25] Briggs KK , Lysholm J , Tegner Y , et al . The reliability, validity, and responsiveness of the Lysholm score and tegner activity scale for anterior cruciate ligament injuries of the knee: 25 years later. Am J Sports Med 2009;37:890–7. 10.1177/0363546508330143 19261899

[26] Mohtadi N . Development and validation of the quality of life outcome measure (questionnaire) for chronic anterior Cruciate ligament deficiency. Am J Sports Med 1998;26:350–9. 10.1177/03635465980260030201 9617395

[27] Lafave MR , Hiemstra L , Kerslake S , et al . Validity, reliability, and responsiveness of the anterior cruciate ligament quality of life measure: a continuation of its overall validation. Clin J Sport Med 2017;27:57–63. 10.1097/JSM.0000000000000292 26780255

[28] Cooperman JM , Riddle DL , Rothstein JM . Reliability and validity of judgments of the integrity of the anterior cruciate ligament of the knee using the Lachman’s test. Phys Ther 1990;70:225–33. 10.1093/ptj/70.4.225 2315385

[29] Galway HR , MacIntosh DL . The lateral pivot shift: a symptom and sign of anterior cruciate ligament insufficiency. Clin Orthop Relat Res 1980;1980:45–50. 7371314

