# Supplementary material for: Healing of acute anterior cruciate ligament rupture on MRI and outcomes following non-surgical management with the Cross Bracing Protocol
Source: Br J Sports Med. 2023 Jun 14;57(23):1490–7. doi: 10.1136/bjsports-2023-106931 (PMC10715498; doi:10.1136/bjsports-2023-106931)
Supplement: Supplementary data [file bjsports-2023-106931supp001.pdf]

Filbay SR, et al. Br J Sports Med 2023;0:1–8. doi:10.1136/bjsports-2023-106931

Appendix 1. The Cross Bracing Protocol

Week	Brace	Aims	Physiotherapy-supervised rehabilitation
1-4	ROM brace fitted and locked at 90 degrees<br><br>NWB with crutches or mobility scooter	• Maintain knee flexion at 90˚ in brace 24/7<br>• Minimise local and global muscle atrophy<br>• Control swelling and pain<br>• DVT risk mitigation: Hydration, calf pump exercises and anticoagulation (Rivaroxaban 10mg daily)<br>• Counsel re anticipated initial discomfort<br>• No NSAIDs medications; no aspiration/drainage of hemarthrosis	• Manual therapy weekly, maintaining 90˚ knee flexion<br>• Cryotherapy<br><br>Rehabilitation exercises:<br>• Quadriceps and hamstring co-contractions.<br>• Calf Theraband plantarflexion<br>• Hip abduction and extension<br>• Contralateral limb: single leg press, leg extension, hamstring curls, calf raise, glute bridge, core activation.<br>• Upper body strengthening.<br>• Ski Ergometer<br>• Grinder

Filbay SR, et al. Br J Sports Med 2023;0:1–8. doi:10.1136/bjsports-2023-106931

5-6	Week 5 = 60 degrees to 90 degrees<br>Week 6 = 45 degrees to 90 degrees<br>NWB with crutches	• Minimise local and global muscle atrophy<br>• Maintain specified knee ROM<br>• DVT risk mitigation: Hydration, calf pump exercises and anticoagulation (Rivaroxaban 10mg daily).	• Manual therapy and exercises weekly in specified range (brace can come off for physiotherapy).<br>Rehabilitation exercises:<br>• Quadriceps and hamstring co-contractions at varying angles<br>• Continue calf Theraband plantarflexion<br>• Hip abduction and extension with ankle weights or Theraband<br>• Continue Contralateral limb; single leg press, leg extension, hamstring curls, calf raise, glute bridge, core activation<br>• Upper body strengthening.<br>• Ski Ergometer<br>• Grinder
7-8	Week 7 = 30 degrees to FULL FLEXION˚<br>Week 8 = 20 degrees to FULL FLEXION<br>PWB with crutches	• Minimise local and global muscle atrophy<br>• Maintain specified knee ROM<br>• DVT risk mitigation: Hydration, calf pump exercises and anticoagulation (Rivaroxaban 10mg daily).	• Manual therapy and exercises weekly in specified range;<br>• Gait retraining.<br>Rehabilitation exercises, as per week 5-6 plus;<br>• Wall squats/holds (Week 7=45˚, Week 8=30˚).<br>• Body weight squats within brace limits
9	Week 9 = 10 degrees to FULL FLEXION.	• Increase knee ROM<br>• Overcome anticipated fixed flexion contracture	• Manual therapy and exercises weekly in specified range.<br>• Gait retraining.<br>Rehabilitation exercises:

Filbay SR, et al. Br J Sports Med 2023;0:1–8. doi:10.1136/bjsports-2023-106931

	Week 10 =<br>UNRESTRICTED ROM brace (0-120˚+)<br>Wean off crutches. Full weight bearing	• Increase muscle strength of lower limbs<br>• Improve proprioception<br>• Cease Rivaroxaban anti-coagulation	• Knee range of motion exercises (heel slides).<br>• Pilates reformer/ leg press<br>• Body weight squats<br>• Bridges - hamstring and gluteal<br>• Crab walks/ monster walks<br>• Calf raises<br>• Static balance exercises<br>• Exercise bike if able to achieve over 100˚ flexion.<br>• Upper body strengthening.<br>• Grinder
10-12	Unrestricted ROM brace<br>Full weight bearing<br>End of week 12:<br>Medical consultation and 12-week MRI.	• Increase knee ROM<br>• Increase muscle strength<br>• Improve proprioception	• Weekly manual therapy and exercises.<br>• Gait retraining.<br>Rehabilitation exercises, as per week 9 plus;<br>• Leg press (single leg)<br>• Hamstring curl machine<br>• Standing and seated calf raise machine<br>• Static balance exercises.<br>• Exercise bike
13-14	Week 13 = Removal of brace. Formal clinical review and repeat MRI.	• Increase muscle strength<br>• Improve proprioception	• Weekly manual therapy and exercises.<br>• Gait retraining.<br>Rehabilitation exercises:<br>• Continue/progress hamstring and quadriceps strengthening<br>• Addition of single leg squatting/Bulgarian squats<br>• Lunges (half range)<br>• Dynamic balance exercises

Filbay SR, et al. Br J Sports Med 2023;0:1–8. doi:10.1136/bjsports-2023-106931

	Full weight bearing<br><br>Brace is worn in high risk environments from week 13-16		• Jogging on spot/mini tramp<br>• Light Skipping<br>• Exercise bike<br>• Upper body<br>• Grinder
15-16	No brace, full weight bearing.	• Increase muscle strength<br>• Improve proprioception<br>• Introduce Running (must meet return to running criteria)	• Manual therapy<br>• Running analysis<br><br>Criteria for commencing slow running:<br>• No effusion<br>• Full ROM<br>• Quadriceps and hamstring strength >80% LSI<br>• Star excursion balance test >90% LSI<br><br>Rehabilitation exercises:<br>Continue/progress previous strength and dynamic balance exercises.<br>• Addition of walking lateral movements e.g. crab walking, side stepping, grapevines etc.<br>• Addition of jumping/landing<br>• Exercise bike<br>• Swimming<br>• Cross trainer/elliptical<br>• Grinder

Filbay SR, et al. Br J Sports Med 2023;0:1–8. doi:10.1136/bjsports-2023-106931

			• Straight line running only (if meeting above criteria)
17	No brace, full weight bearing.	• Increase muscle strength<br>• Improve proprioception<br>• Introduce faster running	• Manual therapy as required<br><br>Criteria for progression to faster running:<br>• Completion of slower running with no pain or effusion<br><br>Rehabilitation exercises:<br>• Continue/progress previous exercises<br>• Introduce faster running (if meeting above criteria)
18	No brace, full weight bearing.	• Increase muscle strength/ proprioception<br>• Introduce Agility exercises	• Manual therapy as required<br><br>Criteria for commencing agility exercises:<br>• Adequate control with jumping and landing, double and single leg<br>• Completion of faster straight line running with no pain or effusion.<br><br>Rehabilitation exercises:<br>• Continue/progress previous exercises<br>• Introduce agility exercises (if meeting above criteria)
19	No brace, full weight bearing.	• Increase muscle strength/ proprioception<br>• Introduce hopping	• Manual therapy as required<br><br>Rehabilitation exercises:<br>• Continue/progress previous exercises<br>• Introduce hopping exercises
20	No brace, full weight bearing.	• Increase and maintain muscle strength/ proprioception	• Manual therapy as required

Filbay SR, et al. Br J Sports Med 2023;0:1–8. doi:10.1136/bjsports-2023-106931

		• Consider return to training from week 20 onwards	Criteria for return to training<br>• Single leg hop >90% LSI<br>• Knee extension strength >90% LSI<br>• Hamstring strength >90% LSI<br>• Good dynamic knee control on single leg land from step<br>• Star excursion balance test >90% LSI<br><br>Rehabilitation exercises:<br>• Continue/progress previous exercises<br>• Return to training (if meeting above criteria)
26+	No brace, full weight bearing.	• Increase and maintain muscle strength/ proprioception<br><br>• Consider return to sport around 9-12 months (the decision to return to sport is goal and criteria based)	• Manual therapy as required<br><br>Criteria for return to play<br>• Triple Hop/lateral hop/ single hop >90% LSI<br>• Single leg press 1.8x BW<br>• Star excursion balance test >90% LSI<br><br>Rehabilitation exercises:<br>• Continue/progress previous exercises<br>Return to play (if meeting above criteria)

ROM: Range of movement, NSAIDs: Non steroidal anti inflammatory drugs, N/PWB: Non/ Partial weight bearing, DVT: Deep vein thrombosis, LSI: Limb symmetry index; BW: Body weight.
