# Supplementary material for: Healing of acute anterior cruciate ligament rupture on MRI and outcomes following non-surgical management with the Cross Bracing Protocol
Source: Br J Sports Med. 2023 Jun 14;57(23):1490–7. doi: 10.1136/bjsports-2023-106931 (PMC10715498; doi:10.1136/bjsports-2023-106931)
Supplement: Supplementary data [file bjsports-2023-106931supp002.pdf]

Appendix 2. Participant outcomes stratified by healing status, excluding n=7 participants who re-ruptured their ACL prior to completing outcomes

	All participants (n=73)	Evidence of ACL healing on 3 month MRI		
		ACLOAS Grade 1 (n=39)	ACLOAS Grade 2 (n=23)	ACLOAS Grade 3 (n=8)
Lysholm Scale score	95 (90 to 100)	99 (94 to 100)	95 (85 to 100)	95 (87 to 100)
ACL-QOL score (Total)	82 (70 to 94)	89 (78 to 96)	71 (61 to 82)	76 (66 to 95)
Symptoms / Physical Complaints	94 (86 to 98)	96 (92 to 100)	88 (82 to 96)	88 (75 to 96)
Work related concerns	100 (93 to 100)	100 (98 to 100)	93 (78 to 98)	100 (98 to 100)
Rec and sport participation	76 (58 to 93)	82 (66 to 93)	61 (42 to 80)	65 (44 to 90)
Lifestyle	90 (77 to 98)	95 (88 to 100)	78 (60 to 93)	88 (73 to 98)
Social and emotional	81 (60 to 94)	90 (76 to 94)	72 (52 to 84)	73 (63 to 97)

For n=2 who did not have an MRI at 3 months, 6 month MRI results are reported (93% of participants had the same ACLOAS score at 3 and 6 months)
