# Supplementary material for: Healing of acute anterior cruciate ligament rupture on MRI and outcomes following non-surgical management with the Cross Bracing Protocol
Source: Br J Sports Med. 2023 Jun 14;57(23):1490–7. doi: 10.1136/bjsports-2023-106931 (PMC10715498; doi:10.1136/bjsports-2023-106931)
Supplement: Supplementary data [file bjsports-2023-106931supp003.pdf]

Appendix 3. Participant characteristics and outcomes reported separately for people with an ACLOAS Grade 2 and 3, on 3 month MRI

Table 1: Participant characteristics

		Evidence of ACL healing on 3-month MRI		
	All participants (n=80)	ACLOAS Grade 1 (n=40)	ACLOAS Grade 2 (n=32)	ACLOAS Grade 3 (n=8)
Age at injury	26 SD 10	27 SD 10	23 SD 9	28 SD 16
Sex (% female)	31 (38)	14 (35)	13 (41)	3 (38)
Time from injury to brace (days)	8 SD 7	6 SD 4	11 SD 8	21 SD 49
Used private health insurance	55 (68)	27 (68)	23 (72)	4 (50)
Level of pre-injury sport				
Recreational	28 (35)	13 (33)	11 (34)	4 (50)
Competitive	49 (61)	25 (63)	20 (63)	3 (38)
Professional	4 (5)	2 (5)	1 (3)	1 (13)
Contact mechanism of injury	24 (30)	17 (43)	5 (16)	2 (22)
History of contralateral ACL injury	8 (10)	3 (8)	4 (13)	1 (13)
Adherent to bracing protocol	77 (95)	37 (93)	31 (97)	8 (100)
PRP injection	14 (17)	5 (13)	8 (25)	1 (13)
Concomitant injuries				
MCL injury	40 (49)	17 (43)	17 (53)	6 (75)
Meniscal injury	39 (48)	21 (53)	12 (38)	6 (75)
PLC injury	31 (38)	20 (50)	5 (16)	6 (75)
Bone contusion	74 (91)	38 (95)	27 (84)	8 (100)
Chondral injury	1 (1)	1 (3)	0 (0)	0 (0)
Sub-cortical fracture	6 (7)	3 (8)	3 (9)	0 (0)
ACL rupture characteristics				
ACL femoral origin intact:				
Lateral displacement of distal remnant	8 (10)	8 (20)	0 (0)	0 (0)
Anterior displacement of distal remnant	9 (11)	6 (15)	2 (6)	0 (0)
No displacement of distal remnant*	19 (24)	19 (48)	1 (3)	0 (0)
Partial avulsion of femoral origin:				
Lateral displacement of distal remnant	10 (12)	1 (3)	7 (22)	2 (25)
Anterior displacement of distal remnant	23 (29)	3 (8)	15 (47)	5 (63)
No displacement of distal remnant*	11 (14)	3 (8)	7 (22)	1 (13)
Complete avulsion of femoral origin	0 (0)	0 (0)	0 (0)	0 (0)

Data are reported as mean SD (standard deviation) or count (proportion);
For the 2 participants with missing 3 month MRI data, ACLOAS scores were estimated using ACLOAS scores form 6 month MRI (70 out of 75 (93%) participants with 6 months MRI data had the same ACLOAS score at 3 and 6 months)
*ACL distal remnant remains inside the boundaries of the intercondylar notch;
PRP=platelet-rich plasma; MCL=medial collateral ligament; PLC=posterior lateral corner;

Table 2. Participant outcomes

		Evidence of ACL healing on 3 month MRI		
	All participants (n=80)	ACLOAS Grade 1 (n=40)	ACLOAS Grade 2 (n=32)	ACLOAS Grade 3 (n=8)
Lysholm Scale score	95 (89 to 100)	98 (94 to 100)	91 (81 to 100)	95 (87 to 100)
ACL-QOL score (total)	80 (69 to 93)	89 (76 to 96)	70 (57 to 76)	76 (66 to 95)
Symptoms / physical complaints	94 (86 to 98)	96 (92 to 100)	88 (82 to 96)	88 (75 to 96)
Work related concerns	98 (90 to 100)	100 (98 to 100)	93 (75 to 99)	100 (98 to 100)
Rec and sport participation	68 (54 to 91)	82 (65 to 93)	58 (38 to 73)	65 (44 to 90)
Lifestyle	88 (74 to 98)	94 (86 to 100)	77 (59 to 88)	88 (73 to 98)
Social and emotional	78 (58 to 94)	90 (72 to 94)	60 (44 to 79)	73 (63 to 97)
3 month Lachman test				
No SSD	64 (80)	40 (100)	23 (72)	1 (12.5)
End point with SSD	15 (19)	0 (0)	9 (28)	6 (75)
No endpoint	1 (1)	0 (0)	0 (0)	1 (12.5)
6 month pivot-shift test				
Normal	44 (58)	33 (85)	11 (37)	0 (0)
Glide	31 (41)	6 (15)	18 (60)	7 (100)
Jerk	1 (1)	0 (0)	1 (3)	0 (0)
Missing, n	4	1	2	1
Return to pre-injury level of sport at 12 months				
Yes	59 (79)	36 (92)	20 (71)	3 (37.5)
No	16 (21)	3 (8)	8 (29)	5 (62.5)
N/A (crossed-over to ACLR before eligible to RTS)	n=5	n=1	n=4	n=0
Underwent arthroscopic surgery	2 (3)	1 (3)	1 (3)	0 (0)
ACL re-rupture	11 (14)	4 (10)	7 (22)	N/A

For n=2 who did not have an MRI at 3 months, 6 month MRI results are reported (93% of participants had the same ACLOAS score at 3 and 6 months)
Missing data from n=1 for the Lysholm Scale and ACL-QOL (ACLOAS Grade 2)
Non-normally distributed data are presented as median (IQR)
SSD=side to side difference; N/A=not applicable; ACLR=anterior cruciate ligament reconstruction; RTS =return to sport
