# Supplementary material for: Healing of acute anterior cruciate ligament rupture on MRI and outcomes following non-surgical management with the Cross Bracing Protocol
Source: Br J Sports Med. 2023 Jun 14;57(23):1490–7. doi: 10.1136/bjsports-2023-106931 (PMC10715498; doi:10.1136/bjsports-2023-106931)
Supplement: Supplementary data [file bjsports-2023-106931supp004.pdf]

Appendix 4. ACLOAS Grades on MRI at 3 and 6-month follow-up (complete case analysis)

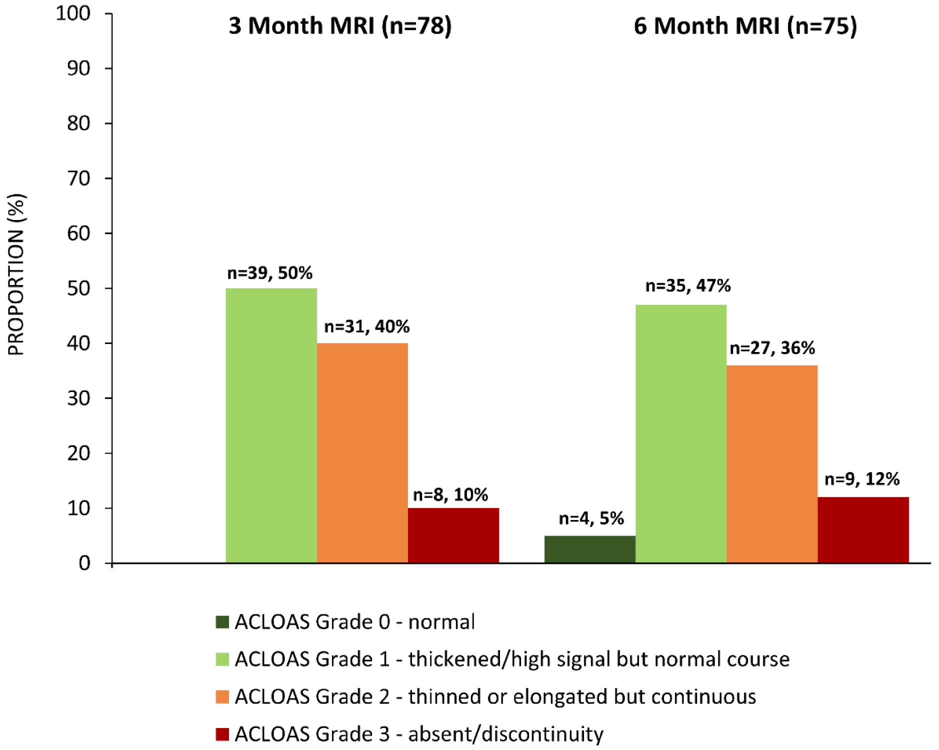

Figure 1: MRI evidence of ACL healing at 3- and 6-month follow-up
There is missing data from n=2 at 3 months (decided not to undergo MRI, n=2), and n=6 at 6 months (due to ACL re-rupture (n=3), pregnancy (n=1), or decided not to undergo MRI (n=2))
