# Supplementary material for: Healing of acute anterior cruciate ligament rupture on MRI and outcomes following non-surgical management with the Cross Bracing Protocol
Source: Br J Sports Med. 2023 Jun 14;57(23):1490–7. doi: 10.1136/bjsports-2023-106931 (PMC10715498; doi:10.1136/bjsports-2023-106931)
Supplement: Supplementary data [file bjsports-2023-106931supp005.pdf]

Appendix 5. Patient-reported outcome scores based on time since injury and healing status on 3-month MRI

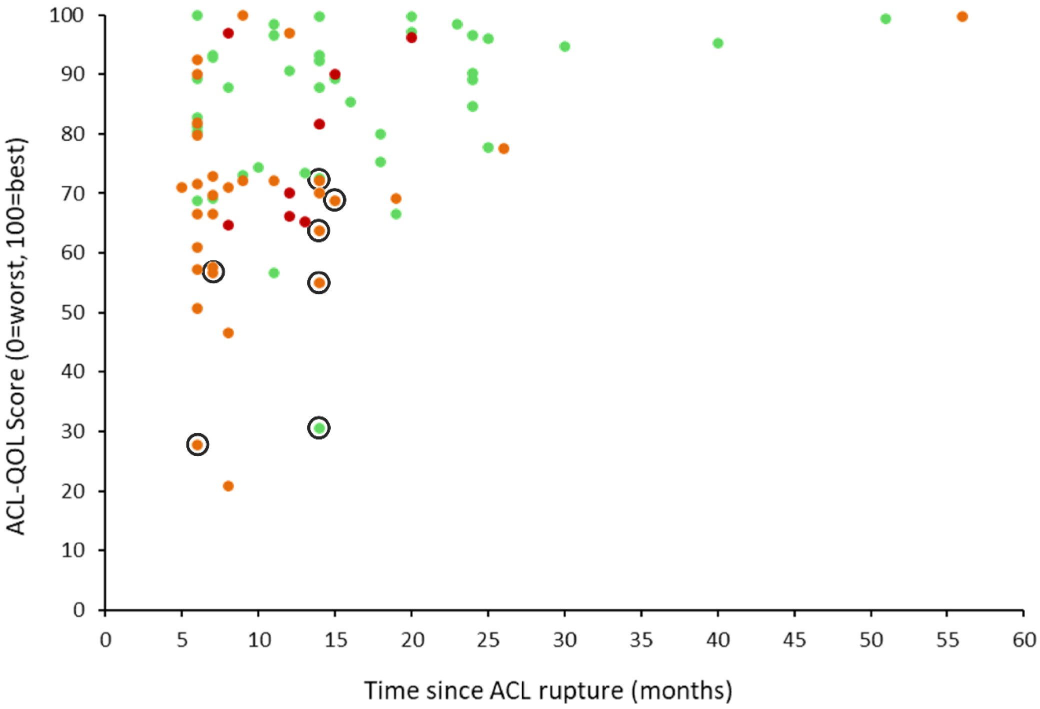

Figure 1. Knee-related quality of life (ACLQOL) depicted over time since ACL rupture and by healing status on 3 month MRI

Green markers = ACLOAS Grade-1 (thickened / high signal but normal course) on 3 month MRI;

Orange markers = ACLOAS Grade-2 (thinned or elongated but continuous) on 3 month MRI;

Red markers = ACLOAS Grade-3 (absent / discontinuity) on 3 month MRI;

Black circles = indicate participants who completed the ACLQOL after re-rupturing their ACL (range: 25 to 286 days after re-injury, n=4 had undergone ACLR 3-4 months before completing the ACLQOL);

There is missing ACLQOL data from 1 participant (ACLOAS score of 2)

The 4 participants who completed patient-reported outcomes 30 to 56 months post-injury, were the first 4 patients to undertake the CBP (the only patients to undertake the CBP between March 2016 and April 2019)

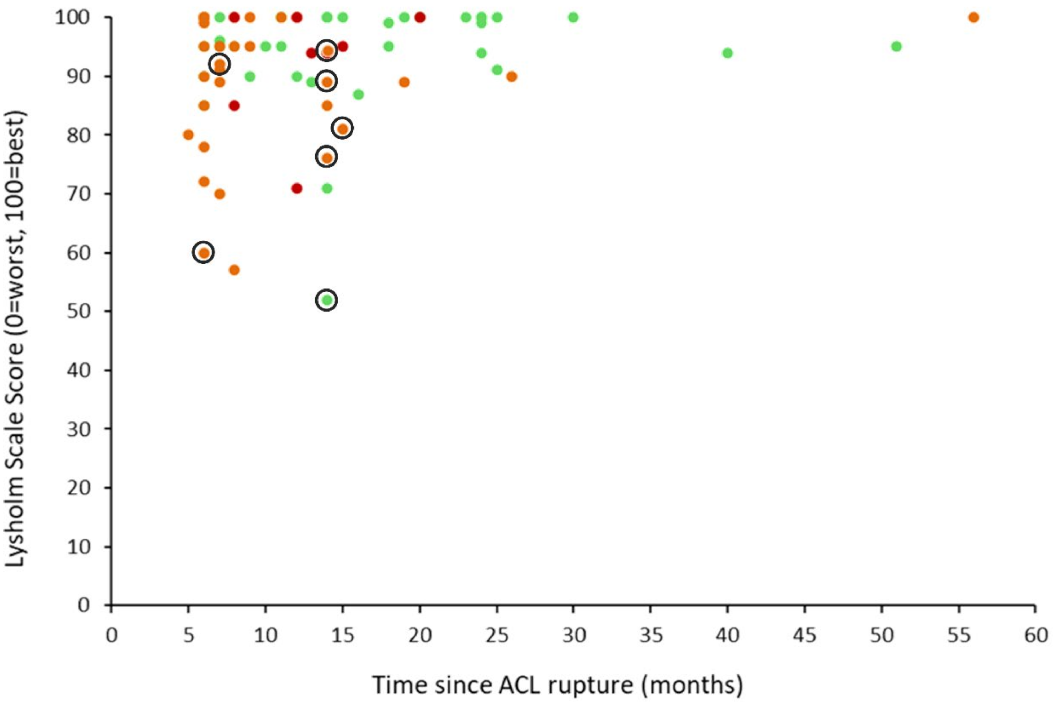

Figure 2. Patient-reported knee function (Lysholm Scale) depicted over time since ACL rupture and by healing status on 3 month MRI

Green markers = ACLOAS Grade-1 (thickened / high signal but normal course) on 3 month MRI;

Orange markers = ACLOAS Grade-2 (thinned or elongated but continuous) on 3 month MRI;

Red markers = ACLOAS Grade-3 (absent / discontinuity) on 3 month MRI;

Black circles = indicate participants who completed the ACLQOL after re-rupturing their ACL (range: 25 to 286 days after re-injury, n=4 had undergone ACLR 3-4 months before completing the ACLQOL);

There is missing Lysholm Scale data from 1 participant (ACLOAS score of 2)

Lysholm Scale scores can be interpreted as “excellent” = 95 to 100 points; “good” = 84 to 94 points, “fair” = 65 to 83 points, or “poor” = less than 65 points;

The 4 participants who completed patient-reported outcomes 30 to 56 months post-injury, were the first 4 patients to undertake the CBP (the only patients to undertake the CBP between March 2016 and April 2019)
